# Intracranial bleeding under vitamin K antagonists or direct oral anticoagulants: results of the RADOA registry

**DOI:** 10.1186/s42466-022-00183-y

**Published:** 2022-05-02

**Authors:** Waltraud Pfeilschifter, Edelgard Lindhoff-Last, Ali Alhashim, Barbara Zydek, Simone Lindau, Stavros Konstantinides, Oliver Grottke, Ulrike Nowak-Göttl, Christian von Heymann, Ingvild Birschmann, Jan Beyer-Westendorf, Patrick Meybohm, Andreas Greinacher, Eva Herrmann, the RADOA-Registry Investigators (Reversal Agent use in patients treated with Direct Oral Anticoagulants or vitamin K antagonists Registry)

**Affiliations:** 1 Department of Neurology and Clinical Neurophysiology, Klinikum Lueneburg, Bögelstr. 1, 21339 Lüneburg, Germany; 2 Pharmazentrum Frankfurt, Institute of General Pharmacology and Toxicology, Goethe University, Frankfurt am Main, Germany; 3 Department of Neurology, University Hospital Frankfurt, Frankfurt, Germany; 4 Coagulation Centre and Coagulation Research Center at the Cardiology Angiology Centre Bethanien Hospital (CCB), Im Prüfling 23, 60389 Frankfurt, Germany; 5 Neurology Department, College of Medicine, Imam Abdulrahman Bin Faisal University, Dammam, Saudi Arabia; 6 Department of Anaesthesiology, Intensive Care Medicine and Pain Therapy, University Hospital Frankfurt, Frankfurt, Germany; 7 Center for Thrombosis and Haemostasis (CTH), Johannes Gutenberg University, Mainz, Germany; 8 Department of Anaesthesiology, RWTH Aachen University Hospital, Aachen, Germany; 9 Institute of Clinical Chemistry, Thrombosis and Haemostasis Treatment Centre, University Hospital, Kiel-Lübeck, Germany; 10 Department of Anaesthesia, Intensive Care Medicine, Emergency Medicine and Pain Therapy, Vivantes Klinikum im Friedrichshain, Berlin, Germany; 11 Institute for Laboratory and Transfusion Medicine, Heart and Diabetes Centre, Ruhr University, Bochum, Germany; 12 Thrombosis Research Unit, Department of Medicine 1; Division Haematology, Dresden University Clinic, Dresden, Germany; 13 Department of Haematology and Oncology, Kings College, London, UK; 14 Department of Anaesthesiology, Intensive Care, Emergency and Pain Medicine, University Hospital Wuerzburg, Wuerzburg, Germany; 15 Department of Immunology and Transfusion Medicine, Universitätsmedizin, Greifswald, Germany; 16 Institute of Biostatistics and Mathematical Modelling, Goethe University, Frankfurt, Germany

## Abstract

**Background and purpose:**

The use of direct oral anticoagulants (DOAC) has increased sharply and DOAC are the oral anticoagulant therapy (OAT) of choice for the majority of patients with newly-diagnosed atrial fibrillation. Intracranial hemorrhage is the most severe adverse event of OAT. Systematic data on the course of intracranial hemorrhage under DOAC compared to vitamin K antagonists (VKA) are warranted to enable shared decision making in AF patients needing OAT.

**Methods:**

This is a secondary analysis of the patients with intracranial bleedings from the prospective multicenter emergency department-based RADOA registry, which collected data on patients admitted with major bleeding while taking VKA or DOAC. The primary endpoint was in-hospital mortality until day 30. We evaluated hematoma volume and short-term clinical outcomes in relation to the extent of active OAT according to coagulation parameters and OAT plasma levels measured by UPLC-MS/MS.

**Results:**

Of 193 patients with major bleeding, 109 (56.5%) had intracranial hemorrhage [52.3% intracerebral (ICH), 33.9% subdural (SDH), 11.0% subarachnoidal (SAH)]. 64 (58.7%) were on VKA and 45 (41.2%) were on DOAC. On admission, we could confirm active anticoagulation in 97.7% of VKA-treated patients based on either INR > 1.3 or phenprocoumon levels and in 75.8% of DOAC-treated patients based on DOAC levels. Patients suffering an intracranial hemorrhage under VKA showed significantly larger hematoma volumes and a higher in-hospital mortality. Especially in intracerebral hemorrhage, we observed a higher initial severity and numerically greater proportion of early changes towards palliative therapy under VKA, which coincided with a numerically higher case fatality.

**Conclusions:**

We show significantly smaller hematoma volumes for ICH and SDH under DOAC in comparison to VKA and a significantly lower 30-day in-hospital mortality rate of DOAC-ICH, even before the introduction of specific antidotes. These data strongly support the use of DOAC whenever possible in patients requiring OAT.

*Trial Registration*: http://www.clinicaltrials.gov; Unique identifier: NCT01722786.

## Introduction

The large phase III trials leading to the approval of the direct oral anticoagulants (DOAC) unanimously showed that direct oral anticoagulant (DOAC) treatment was associated with a lower rate of hemorrhagic stroke than treatment with vitamin K antagonists (VKA) while being at least equally effective in preventing ischemic stroke in patients with AF [1]. But while intracranial hemorrhage as a severe complication of long-term anticoagulation was less frequent, the mortality rate did not differ significantly between both OAT regimes in those trials [2, 3]. Since then, several real life analyses of large health insurance databanks have confirmed a favorable safety profile of DOAC in comparison to VKA with regard to mortality, ischemic events and hemorrhagic stroke [4]. Studies on the management and clinical course of intracranial hemorrhage under oral anticoagulant therapy (OAT) that detail hematoma volume, hematoma expansion, coagulation parameters and reversal strategies under oral anticoagulant therapy (OAT) are mostly retrospective [5, 6] or report patients preselected by clinical field (e.g. registries from stroke units or neurological intensive care units) [7, 8] and hence are vulnerable to different types of biases. A meta-analysis of 19 trials comparing haematoma volume, hematoma expansion, and mortality in VKA-ICH vs. non-OAT-ICH established a more unfavourable course of all these parameters in VKA-ICH patients [7]. Concerning differences between the different OAT regimens, individual studies so far yielded conflicting results concerning hematoma volumes and expansion as well as outcome of the two most common bleeding entities, intracerebral hemorrhage (ICH) [8, 9] and subdural hematoma (SDH) [10, 11] under DOAC vs. VKA. Our prospective multicenter registry, which enrolled patients at the level of the emergency departments (ED), adds a well-defined cohort characterized by a virtually complete capture of patients with intracranial hemorrhage even in the case of an early decision to pursue palliative therapy with a laboratory-aided acquisition of patients’ coagulation status and reversal strategies.

## Methods

### RADOA registry

The design of the prospective, observational, non-interventional, open-label, investigator-initiated multicenter German registry of severe hemorrhage under OAT has been described previously in more detail [12, 13]. All 10 study centers had to document efforts directed at consecutive enrollment via a screening log and were subject to 100% independent on site data monitoring. The study protocol was approved by the institutional ethics committee (IEC) of Frankfurt University Hospital with secondary approvals by all relevant IECs. Primary endpoint in the registry was in-hospital mortality up to day 30.

### Patients

Patients on OAT with severe hemorrhage and/or urgent interventions were enrolled at the level of the ED and the clinical course and outcome were prospectively documented up to 30 days. For this analysis, all patients with intracranial hemorrhage were selected.

### ICH and SDH volumetry

A board-certified neurologist (AAH) blinded to all other patient data centrally quantified hematoma volume using the ABC/2 method, which is well-established for ICH [14] but also validated for SDH [15]. Hematoma expansion was defined as an increase in volume > 12.5 mL or 33% volume increase.

### Coagulation analyses and definition of ‘active OAT’

Routine laboratory parameters including hematology, INR/PT, and aPTT were recorded on admission and during the course of in-hospital stay. Concentrations of DOACs and VKA were measured in back-up blood samples from routine care at the Institute for Laboratory and Transfusion Medicine, Heart and Diabetes Centre, Ruhr University, Bochum, Germany using UPLC-MS/MS analysis.

Active OAT in case of VKA was defined as an INR > 1.3 and/or a phenprocoumon plasma level of > 0.2 mg/L. In case of DOAC, a plasma DOAC level of > 30 ng/mL and/or a last intake < 12 h for apixaban and < 24 h for rivaroxaban and edoxaban was required. Four patients without DOAC level quantification and without known last intake were classified as OAT-negative.

### Statistical analysis

Quantitative variables are reported as mean ± standard deviation and are compared between groups with nonparametric Wilcoxon Mann Whitney test. Categorical variables are reported as counts and rates in percent and compared between groups by the exact Fisher test. Kaplan Meier curves are used for illustrating and log-rank tests are used for comparing 30 days in hospital survival data, respectively. All statistical tests were two-sided and used a significance level of alpha = 5%.

## Results

**Fig. 1:**
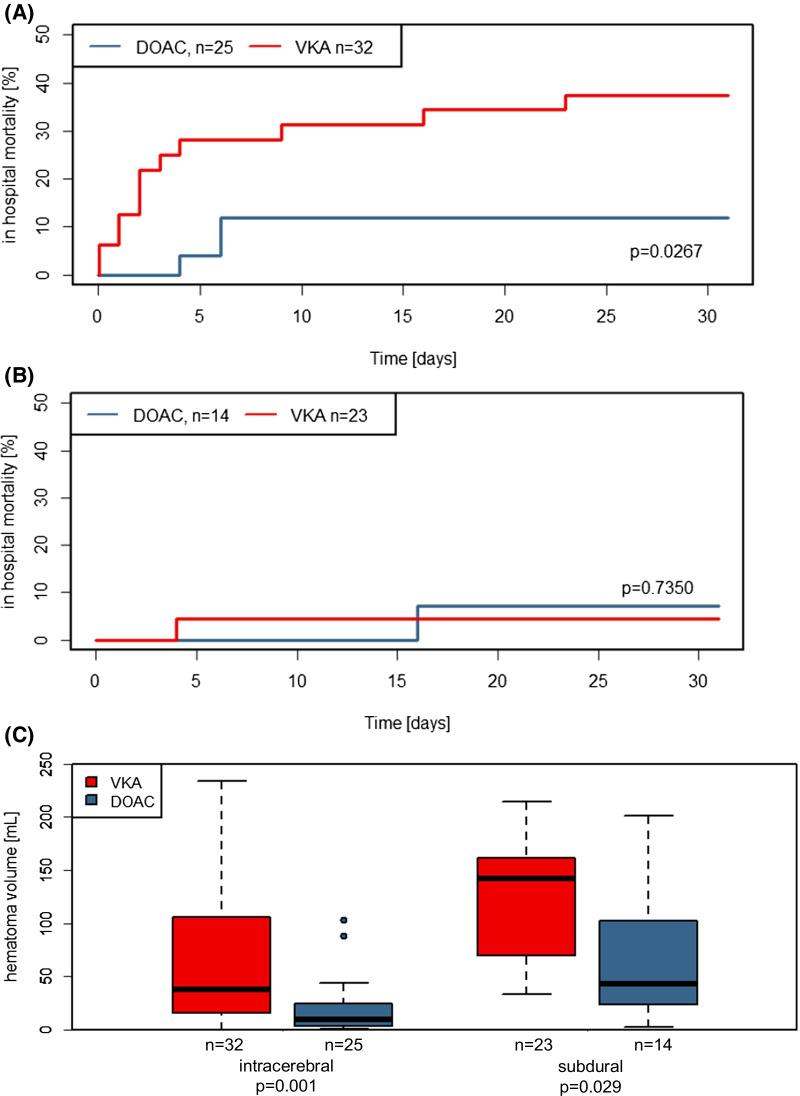
Panel A and B: 30-day in-hospital mortality and hematoma volume of VKA- and DOAC-associated ICH and SDH. Kaplan Meier curves and p-values from log-rank test for 30 day in-hospital mortality in patients with intracerebral (**A**) and subdural (**B**) hemorrhage. Compared are patients treated with vitamin K antagonists and direct oral anticoagulants at hospital admission. Panel (**C**) Boxplots comparing hematoma volume in patients with intracerebral and subdural hemorrhage treated with vitamin K antagonist (VKA) and direct oral anticoagulants (DOAC) at hospital admission. As usual, horizontal lines represent the median of the corresponding subgroups. P-values are from a two-sided nonparametric Wilcoxon–Mann–Whitney U-test

Among 193 patients with major bleeding, 109 (56.5%) had intracranial hemorrhage (52.3% intracerebral [ICH], 33.9% subdural [SDH], 11.0% subarachnoidal [SAH]). 64 (58.7%) were on VKA and 45 (41.2%) were on DOAC. The respective share of VKA and DOAC did not differ significantly between bleeding locations and the distribution of age and sex, physiological variables and risk factors for AF-associated stroke (CHA_2_DS_2_-VASc score) and bleeding under OAT (HAS-BLED score) was well-balanced between patients on VKA and DOAC (Table 1). Notably, 35.7% of intracranial hemorrhages were related to a reported fall or accident (Table 2). Focusing on ICH and SDH as the two most frequent types of intracranial bleeding with quantifiable hematoma volumes, we found concordantly greater hematoma volumes in patients on VKA compared with DOAC (ICH: 63.8 mL ± 59.3 vs. 20.7 mL ± 26.6; p = 0.001; SDH: 125 mL ± 58.5 vs. 69.7 mL ± 61.2; p = 0.029, Table 2 and Fig. 1C) with a numerically larger proportion of intraventricular hemorrhage in ICH under VKA, whereas small hematoma (< 30 mL) was more frequent in ICH and SDH under DOAC. In ICH patients, we found equal rates of ‘no hematoma expansion’ (55% in DOAC-ICH vs 56% in VKA-ICH) but follow-up imaging was unavailable in a significantly larger share of VKA-ICH patients (24% vs. 0% of DOAC-ICH). This corresponded to a higher proportion of patients with VKA-ICH being switched to palliative care in the first 24 h as well as during the course of the hospital stay. In SDH, we found a higher rate of hematoma expansion in DOAC-SDH as compared to VKA-SDH against a background of significantly smaller hematomas on first imaging and there was no significant difference of mortality between the two groups in SDH patients, who overall showed a lower 30 day in-hospital mortality compared to ICH patients (Table 2, Fig. 1B).

**Table 1:** Patient characteristics by OAT and intracranial bleeding location

	VKA-ICH n = 32	DOAC-ICH n = 25	p-value	VKA-SDH n = 23	DOAC-SDH n = 14	p-value
Male sex	18 (56.2%)	12 (48.0%)	p = 0.5994	16 (69.6%)	11 (78.6%)	p = 0.7099
Age	76.0 ± 13.4	78.5 ± 8.4	p = 0.5194	76.6 ± 10.4	82.1 ± 6.4	p = 0.1578
Age > 65 y	28 (87.5%)	23 (92.0%)	p = 0.6856	19 (82.6%)	14 (100%)	p = 0.2760
Height [m]	1.7 ± 0.1	1.7 ± 0.1	p = 0.3717	1.7 ± 0.1	1.7 ± 0.1	p = 0.8189
Weight [kg]	84.4 ± 16.0	76.9 ± 15.3	p = 0.0544	82.0 ± 25.6	83.4 ± 10.5	p = 0.3979
BMI [kg/m^2^]	28.1 ± 5.5	26.3 ± 5.1	p = 0.0619	26.7 ± 6.9	27.6 ± 5.2	p = 0.5700
Antiplatelet drugs	2 (6.2%)	2 (8.0%)	p = 1.0000	7 (30.4%)	4 (28.6%)	p = 1.0000
Indication						
Non-valvular arterial fibrillation	22 (68.8%)	20 (87.0%)	p = 0.3805	19 (82.6%)	11 (78.6%)	p = 1.000
Deep vein thrombosis	3 (9.4%)	0 (0.0%)	p = 0.2481	1 (4.3%)	1 (7.1%)	p = 1.0000
Other or unknown	7 (21.9%)	5 (20,0%)	p = 1.0000	3 (13.0%)	2 (14.3%)	p = 1.000
CHADS-VASC Score	4.7 ± 1.7	4.3 ± 1.6	p = 0.4736	4.9 ± 2.0	4.4 ± 1.1	p = 0.2893
HAS-BLED Score	2.8 ± 0.8	2.6 ± 1.1	p = 0.6929	2.5 ± 1.3	2.8 ± 1.0	p = 0.7086
HAS-BLED Score modified	2.7 ± 0.9	2.5 ± 1.0	p = 0.6616	2.4 ± 1.1	2.8 ± 1.0	p = 0.4513
Treatment						
Apixaban		10 (40%)			8 (57.1%)	
Edoxaban					1 (7.1%)	
Rivaroxaban		15 (60%)			5 (35.7%)	
Phenprocoumon	32			23		

Data are presented as n (%) or mean ± SD

**Table 2:** Clinical course by OAT and intracranial bleeding location

	VKA-ICH n = 32	DOAC-ICH n = 25	p-value	VKA-SDH n = 23	DOAC-SDH n = 14	p-value
Bleeding after fall or likely fall	6 (18.8%)	4 (16.0%)	p = 1.0000	11 (47.8%)	8 (57.1%)	p = 0.7374
Loss of consciousness	16 (50.0%)	5 (20.0%)	p = 0.0275	5 (21.7%)	3 (21.4%)	p = 1.0000
Mechanical ventilation	13 (40.6%)	6 (24.0%)	p = 0.2597	5 (21.7%)	4 (28.6%)	p = 0.7046
Acute renal failure	0 (0.0%)	1 (4.0%)	p = 0.4386	1 (4.3%)	1 (7.1%)	p = 1.0000
Systolic BP on admission	142 ± 35.5	166 ± 48.7	p = 0.0598	145 ± 24.8	160 ± 24.3	p = 0.1714
Effective anticoagulation on admission^a^	32 (100%)	21 (84.0%)	p = 0.0320	23 (100%)	12 (85.7%)	p = 0.1366
Hematoma volume (mL)	63.8 ± 59.3	20.7 ± 26.6	p = 0.0013	125 ± 58.5	69.7 ± 61.2	p = 0.0287
Small hematoma (< 30 mL)	12 (41.4%)	19 (79.2%)	p = 0.0109	0 (0.0%)	3 (30.0%)	p = 0.0410
Intraventricular hematoma	17 (60.7%)	10 (43.5%)	p = 0.2672			
Hematoma expansion (< 24 h)						
No	14 (56.0%)	11 (55.0%)	p = 1.0000	13 (100%)	5 (55.6%)	p = 0.0172
Yes	5 (20.0%)	9 (45.0%)	p = 0.0265	0 (0.0%)	4 (44.4%)	p = 0.0172
Unknown in palliative patients	6 (24.0)	0 (0.0%)	p = 0.1071	0 (0.0%)	0 (0.0%)	p = 1.0000
Re-bleeding within 30 days (> 24 h)						
No	22 (95.7%)	19 (100%)	p = 1.0000	17 (89.5%)	12 (92.3%)	p = 1.0000
Yes	0 (0.0%)	0 (0.0%)	p = 1.0000	2 (10.5%)	1 (7.7%)	p = 1.0000
Unknown in palliative patients	1 (4.3%)	0 (0.0%)	p = 1.0000	0 (0.0%)	0 (0.0%)	p = 1.0000
PCC application	25 (78.1%)	17 (68.0%)	p = 0.5456	21 (91.3%)	11 (78.6%)	p = 0.3459
Sufficient PCC application (30-50 IE/kgKG as first application)	16 (66.7%)	12 (70.6%)	p = 1.0000	9 (45.0%)	5 (45.5%)	p = 1.0000
Operation at bleeding site	12 (46.2%)	8 (36.4%)	p = 0.5651	16 (76.2%)	7 (50.0%)	p = 0.1534
Re-operation within 30 days	2 (9.1%)	0 (0.0%)	p = 0.4902	2 (11.1%)	1 (7.7%)	p = 1.0000
30 day in-hospital mortality	12 (37.5%)	3 (12.0%)	p = 0.0374	1 (4.3%)	1 (7.1%)	p = 1.0000
Palliative care	6 (18.8%)	2 (8.0%)	p = 0.4444	0 (0.0%)	0 (0.0%)	p = 1.0000

Data are presented as n (%) or mean ± SD

^a^Effective anticoagulation was assumed if the patient had a plasma DOAC level of > 30 ng/ml and/or a last intake of < 12 h for apixaban and < 24 h for rivaroxaban and edoxaban or a phenprocoumon plasma level of > 0.2 mg/l and/or an INR > 1.3

Patients with ICH under VKA were more often comatose, required mechanical ventilation and had a lower initial blood pressure, which could be interpreted as a marker of higher disease severity (e.g. mechanical ventilation requiring analgosedation). We observed a statistically non-significant higher frequency of neurosurgical procedures and a significantly higher 30 day in-hospital mortality in VKA-ICH vs. DOAC-ICH (VKA: 37.5% vs. DOAC: 12.0%; 95% confidence interval for the rate difference from 3 to 44%; p = 0.0374; Table 2, Fig. 1A).

Concerning the level of anticoagulation, we could confirm active anticoagulation in all 55 patients of patients with VKA-associated ICH or SDH by means of either an INR > 1.3 (n = 46/55, 84%) or mass-spectrometry measurements of phenprocoumon levels (n = 43/44, 98%) and in 84.6% of DOAC-associated ICH or SDH by means of mass-spectrometry DOAC level measurements (n = 25/33, 76%) or medical history (n = 14/17, 82%). Notably, we observed a fairly high frequency of four factor prothrombin complex concentrate (PCC) application, which was the only available strategy in patients treated with VKA and with DOACs (solely direct factor Xa inhibitors), during the time of patient accrual. Guideline-recommended doses of 30–50 mg/kg BW were used in approximately 70% of all the reversals in ICH patients but only 45% of SDH patients. No significant difference was observed in 30 day in-hospital mortality between patients who received PCC and patients who did not.

## Discussion

In our prospective multicenter registry enrolling patients with major bleeding under OAT, we found a high proportion of intracranial bleedings. Since patients were enrolled at the level of the emergency department, our population was broader and less selective than other ongoing registry trials that target neurology/neurosurgery departments. This may explain the considerably larger mean hematoma volume in patients (> 60 mL) with VKA-ICH in comparison to these registries [7, 9]. 25% of the VKA-ICH patients did not receive a follow-up CT scan. In a life-threatening situation, this most probably indicates that they were switched to palliative care, which was explicitly documented in 18% of this group. These patients may well be missed by registries that capture patients only after being transferred to specialist NICU or stroke unit care. This could lead to an underestimation of the severity and high mortality of VKA-ICH. By contrast, the mean hematoma volume reported for DOAC-ICH was well in the range of previously published registry data for DOAC-ICH [9]. While VKA-ICH was associated with a high mortality rate of 37.5% and the majority of deaths occurred within the first five days after hospital admission, the mortality rate of DOAC-ICH was significantly lower (12%). SDH under DOAC also showed smaller initial hematoma volumes than SDH under VKA, but the 30-day in-hospital mortality, which was generally lower, did not differ between the two OAT regimens. Most notably, half of the SDH under both OAT regimens were fall-associated, indicating the need for preventive measures.

By virtue of the measurements of phenprocoumon plasma levels, we were able to show that all patients with VKA-ICH were actively anticoagulated at the time of ICH. This differs significantly from other registries that rely on the INR and show rates of apparently subtherapeutic anticoagulation as high as 16% [9]. The inclusion of patients that received PCCs prior to the transport from a primary hospital to a larger center where they were enrolled into the registry after INR normalization may lead to this discrepancy. Determination of DOAC-concentrations also allowed us to confirm active anticoagulation in the majority of DOAC-ICH. The neutral results concerning a significant association of PCC reversal of OAT with a lower 30-day in-hospital mortality should not be overinterpreted due to the small sample size.

## Conclusions

This is the first multicenter registry consecutively enrolling patients with intracranial bleeding at the level of the emergency department circumventing some of the biases of registries enrolling at specialist care level. We show significantly larger hematoma volumes for ICH and SDH under VKA in comparison to DOAC and a significantly higher 30-day in-hospital mortality rate of VKA-ICH. These data strongly support the use of DOAC whenever possible in patients requiring OAT.

## Data Availability

The data are available from the senior author (Prof. Dr. Edelgard Lindhoff-Last) upon request.
